# 195 Falls: Ireland’s Unexpected Leader in Trauma - A Leap Towards Healthier Moves

**DOI:** 10.1093/eurpub/ckae114.013

**Published:** 2024-09-26

**Authors:** Louise Brent, Emer Ahern, Terence Murphy, Conor Deasy

**Affiliations:** National Office Of Clinical Audit, Dublin, Ireland

## Abstract

**Background:**

Falls from a low height i.e. standing height or less are the leading cause of trauma in Ireland. Each year almost 4000 patients fracture their hip and 3000 major trauma patients suffer life threatening or life changing injuries due to a fall.

**Methods:**

The National Office of Clinical Audit manages two national trauma based clinical audits, the Irish Hip Fracture Database since 2012 and the Major Trauma Audit. Combined they have data on over 50,000 patients who have suffered serious injury from a fall. Both audts are web-based and clinically led and contain data on patient demographics, care standards, injury profile and outcomes. This data is reported back to the healthsystem on a continuous basis to facilitate care planning and quality improvment.

**Results:**

95% of hip fractures are attributable to a low fall in the home or nurisng home and 62% of major trauma is from low falls 50% of which was in the home. As age increased so too does the proportion of patients presenting with a fall. Data from the audits shows that older patients are less likely to be suspected of serious injuries due to the inocuous nature of the mechanism of injury and therefore recognition of the serious nature of their injuries is delayed and often patients are not seen as promptly as they should by senior clinicans and outcomes are much worse then those who did not present by a ‘low mechanism injury’. Both audits have demonstrated this is increasing, in line with our ageing population and the protracted time patients spent in their homes during the pandemic has led to a further increase in patients presenting with falls and frailty.

**Conclusion:**

To date education materials have been developed on how to keep safe in the home, falls prevention and home activity advice for healthcare workers, public health and patients. This has generated some coverage in the media but more needs to be done to keep our population fit and active and aware of the importance of bone health to support not just an active life but also safe lifestyle.

